# Improving Rainbow Trout (*Oncorhynchus mykiss*) Juvenile Performance and Intestinal Condition Through Lactic Acid Bacteria Feed Fermentation

**DOI:** 10.3390/ani16101482

**Published:** 2026-05-12

**Authors:** Gustavo Alberto Arbelaez Rojas, Nataly Neves Oliveira dos Santos, Larissa Stockhausen, Bia Pacheco Kozikowski, Mariana Bender, Fernanda Regina Delzivo, Luiz Augusto Cipriani, Everton Skoronski, Pedro Toledo, German Merino Araneda, Thiago El Hadi Perez Fabregat

**Affiliations:** 1 Programa de Pós-Graduação em Ciência Animal, Departamento de Produção Animal e Alimentos, Centro de Ciências Agroveterinárias, Universidade do Estado de Santa Catarina, Av. Luiz de Camões, 2090, Bairro Conta Dinheiro, Lages 88520-000, SC, Brazil; 2 Programa de Postgrado Magíster en Acuicultura, Departamento de Acuicultura, Facultad de Ciencias del Mar, Universidad Católica del Norte, Larrondo 1281, Coquimbo 1781421, Chile; 3 Departamento de Engenharia Ambiental e Sanitária, Centro de Ciências Agroveterinarias, Universidade do Estado de Santa Catarina, Av. Luiz de Camões, 2090, Bairro Conta Dinheiro, Lages 88520-000, SC, Brazil

**Keywords:** fermented feed, intestinal health, lactic acid bacteria, sustainability

## Abstract

This study evaluated the effects of feed fermented with *Lactobacillus acidophilus* and *Lacticaseibacillus rhamnosus* on the growth performance and intestinal condition of the juvenile rainbow trout *Oncorhynchus mykiss*. Three fermentation times (3, 6, and 9 h) were compared against a non-fermented control diet. The experimental design followed a randomized block scheme, with four treatments and four replicates. Juvenile rainbow trout (22.2 ± 0.9 g) were distributed among 16 tanks (500 L) connected to a recirculation system at a density of 15 fish per tank. A commercial diet (45% crude protein) was fermented through solid-state fermentation. After 60 days, growth performance and intestinal condition were assessed. The microbial fermentation of extruded commercial feeds was efficient, increasing the lactic acid bacteria count and reducing pH within 6 h. Feeding fermented feeds reduced feed intake without affecting fish growth performance. Feed fermented for 3 and 6 h improved feed efficiency, and evidence of enhanced nitrogen utilization was observed. In addition, feed fermented for 6 h increased intestinal colonization by lactic acid bacteria and enhanced intestinal lipase activity. Feed fermented for 9 h showed evidence of improved intestinal condition. Overall, this study indicates that feed fermentation improves feed efficiency and intestinal condition in rainbow trout.

## Simple Summary

This study evaluated the effects of feed fermented with *Lactobacillus acidophilus* and *Lacticaseibacillus rhamnosus* on the growth performance and intestinal condition of the juvenile rainbow trout *Oncorhynchus mykiss*. The microbial fermentation of extruded commercial feeds was efficient, increasing the lactic acid bacteria count and reducing pH within 6 h. Feeding fermented feeds reduced feed intake without affecting fish growth performance. Feed fermented for 3 and 6 h improved feed efficiency, and evidence of enhanced nitrogen utilization was observed. In addition, feed fermented for 6 h increased intestinal colonization by lactic acid bacteria and enhanced intestinal lipase activity. Feed fermented for 9 h showed evidence of improved intestinal condition. Overall, this study indicates that feed fermentation improves feed efficiency and intestinal condition in rainbow trout.

## 1. Introduction

Rainbow trout (*Oncorhynchus mykiss*) is one of the most widely produced fish species worldwide [1]. Its meat has high commercial value, and production typically occurs in cold, abundant water resources. Rainbow trout farming is often associated with tourism in well-preserved regions, and production can be conducted with low environmental impacts [2]. Although rainbow trout rearing is feasible on a small scale, feed costs are high, and feed management strongly affects economic profitability [3]. Nutritional requirements and feeding practices for rainbow trout have been extensively investigated [4]. However, improvements in performance indicators such as growth rate, feed conversion efficiency, and overall health are difficult to achieve solely through changes in feed composition. In this context, the feed fermentation represents an innovative application of well-established technology, offering a promising alternative to improve feed conversion efficiency and promote the long-term economic sustainability of rainbow trout aquaculture.

Fermentation is a commonly used technique for enhancing nutritional value and preserving foods [5,6]. More recently, this process has been applied to fish feeds to improve their nutritional quality and confer functional properties to the fish feed [7,8]. During fermentation, nutrients become partially pre-digested, beneficial microorganisms with probiotic potential proliferate, and secondary metabolites, such as organic acids, bacteriocins, exopolysaccharides, and bioactive peptides, are produced [9,10,11,12]. Fermentation can be carried out with select microorganisms capable of lactic acid production, particularly those belonging to the group of lactic acid bacteria [13,14]. Co-fermentation using mixed microbial cultures typically generally lead to better results compared to single-strain fermentation [15]. *Lactobacillus acidophilus* and *Lacticaseibacillus rhamnosus* are widely used strains recognized for their functional benefits [16]. In feed fermentation, *L. acidophilus* has demonstrated positive effects, both alone [8] and in combination with other lactic acid bacteria [7].

The use of fermented feeds in fish feeding has yielded positive results on survival rate [8] and feed conversion efficiency [7]. These improvements are particularly because they directly reduce production costs. In addition, these studies also demonstrated effects on intestinal microbiota composition and overall intestinal health. Although fermented ingredients have shown promising results in salmonids diets [17,18,19], feed fermentation itself remains a relatively new research area, with several unexplored implications. Rapid fermentation may serve as an effective strategy for bioactivating fish feed, potentially enhancing growth performance and intestinal health. This approach is simple, cost-effective, and compatible with extruded feeds. Therefore, the objective of this study was to evaluate whether the fermentation of extruded feed with *Lactobacillus acidophilus* and *Lacticaseibacillus rhamnosus* would improve the zootechnical performance, metabolic profile, and intestinal condition of juvenile rainbow trout.

## 2. Methods

### 2.1. Experimental Design and Ethical Statement

This study was conducted at the Fish Farming Laboratory of Agroveterinary Science Center of the Santa Catarina State University in Brazil. The research was approved by the University’s Ethics Committee on 27 June 2025, under protocol number 8786291024. Three time periods (3, 6 and 9 h) of fermentation of the feed were evaluated in comparison with a control feed without fermentation. The experimental design was in randomized blocks with four treatments and four replicates, and the fish were evaluated over 60 days. The block design was used to isolate the effects of lighting on the tanks.

### 2.2. Feed Fermentation

Commercial extruded feed (Neovia Nutrição e Saúde Animal Ltd., Paulinia, Brazil)—45% crude protein and 4 mm) was acquired from a local supplier. The basic composition of the feed was broken rice, rice bran, soybean meal, wheat bran, meat and bone meal, fish meal, blood meal, poultry by-product meal, and fish oil. The feed was fermented through an adaptation of the solid-state feed fermentation methodology by [8]. Pilot tests were conducted to achieve consistency between the batches. Fermentation was carried out with *Lactobacilus acidophilus* (DMS 21717, Coana^®^, Florianópolis, Brazil) and *Lacticaseibacillus rhamnosus* GG (ATCC 53103, Nutribiota, São Paulo, Brazil). Each strain was individually reactivated and inoculated directly into the feed at a concentration of 8 log CFU g^−1^. The feed was adjusted to 15% moisture using sterile mineral water. The feeds were mixed and arranged in trays, maintaining a maximum height of two centimeters of feed per tray. Fermentation was carried out in a forced-air oven at 36 °C for 12 h to determine the optimal fermentation time. Once the fermentation was complete, the feeds were dried in a forced-air oven (36 °C) until they reached a constant weight (dry matter) and then kept in a freezer (−20 °C). After fermentation, the feeds were ground and sieved to obtain particles of approximately 2 mm in diameter, suitable for fish mouth size.

### 2.3. Fermented Feed Characterization

During fermentation, samples were collected every three hours to determine the lactic acid bacteria count and pH. Based on these results, three fermentation times were selected for bromatological analyses and subsequent testing in fish. For lactic acid bacteria count, 1.6 g of each sample was weighed and homogenized in sterile tubes containing sterilized peptone water for viable cell counting (Log CFU g^−1^). The samples were vortexed for approximately one minute, and serial dilutions were carried out in sterilized glass tubes containing peptone water. Subsequently, 0.1 mL of the chosen dilutions were plated, in triplicate, on MRS agar plates for lactic acid bacteria enumeration. The plates were incubated upside down in an oven at 36 °C for 48 h to count the CFU mL^−1^. For pH, the samples were weighed and diluted in distilled water. The analyses were performed with 5 g samples, which were weighed and diluted in distilled water. The pH was measured with a benchtop pH meter (AKSO, Simpla model pH140, São Leopoldo, Brazil).

The proximal composition [20] and free amino acid composition [21] analyses were performed at the Nutrition Laboratory of the Universidad Católica del Norte. The compositions of the experimental feeds are presented in Table 1 and Table 2.

### 2.4. Animals and Facilities

The unsexed juvenile rainbow trout were acquired from a commercial fish farmer (Piscicultura Basquerote, Bocaina do Sul, Brazil) from in Santa Catarina, Brazil. After a two-week acclimatization period, the fish (22.2 ± 0.90 g) were randomly distributed into 16 tanks (500 L) with a density of 15 fish per tank. The tanks were connected to a recirculation system equipped with mechanics and biological filters. All tanks were equipped with an individual aeration system connected to a radial air compressor. The fish were fed once a day by hand until apparent satiety. This management method was chosen to reduce feed conversion and organic solids in the system [22]. The pellets were ground and sieved to a particle size of 3 mm to allow for ingestion by all fish.

Temperature was measured daily, and the remaining organic matter was removed by siphoning. The dissolved oxygen (Alfakit AT-170, Florianópolis, Brazil), pH (HI98130, Hanna Instruments, Brazil), salinity (AKSO, AK87, São Leopoldo, Brazil), and total ammonia (LabconTest, Alcon Pet, Camboriú, Brazil) were measured once a week. The water in the tanks was slightly salinized (2 g L^−1^) to reduce susceptibility to disease. The mean values of the water quality variables during the experiment were maintained as follows: temperature of 16.3 ± 4.6 °C, dissolved oxygen of 7.55 ± 0.86 mg L^−1^, pH of 8.54 ± 0.31, salinity (ppt) of 2.15 ± 0.72, and total ammonia of 0.41 ± 0.31 mg L^−1^.

### 2.5. Fish Performance

At the beginning and end of the experiment, all fish were fasted for 24 h, anesthetized with eugenol (40 mg L^−1^) [23], and weighed individually. The following productive performance parameters were evaluated: final weight, daily weight gain (DWG = (final average weight − initial average weight)/experimental period), feed conversion rate (FCR = feed intake/total weight gain), specific growth rate (SGR = ((average final weight − average initial weight)/experimental period) × 100), hepatosomatic index (HSI = (liver weight)/(total fish weight) × 100) and survival rate (S = (total animals harvested/total animals stocked* 100)).

At the end of the experimental period, after weighing, eight animals from each experimental unit were anesthetized and euthanized for the collection of biological samples. Two animals per experimental unit were used for each of the analyses described below.

### 2.6. Hepatic and Muscular Ammonia

Samples of white muscle and liver were deproteinized in 1 mL of trichloroacetic acid (TCA 20%), processed in a mechanical homogenizer, and centrifuged (Loccus, refrigerated centrifuge model L3024, Cotia, Brazil) at 12,000× *g* for 3 min at 4 °C. Aliquots of the supernatant in an appropriate volume were transferred to Eppendorf tubes and completed with distilled water to a final volume of 2 mL, to which 0.5 mL of Nessler reagent (Metaquimica, Jaraguá do Sul, Brazil) was added, with incubation at room temperature for 20 min.

The samples were then read on a spectrophotometer (Thermoscientific—Genesys 150, Waltham, MA, USA) at an absorbance at 420 nm and compared with a standard of known concentration (100 nmoles) of ammonium chloride (NH_4_Cl) [24]. It was necessary to perform the ammonia calibration curve with the Nessler reagent and standardization with the tissues, white muscle, and liver. The final ammonia concentration was expressed in µmoles per mg of tissue.

### 2.7. Intestinal Microorganism Count

The intestines were aseptically removed, weighed to 0.1 g, minced, homogenized, and serially diluted (1:10) in test tubes containing sterile saline (0.65%). The intestinal homogenates were then plated on Petri dishes with MRS agar (Man Rogosa Sharpe agar), TSA agar (tryptone soy agar), and TCBS agar for acid lactic bacteria, heterotrophic bacteria, and *Vibrio* sp., respectively. The intestinal homogenates seeded in Petri dishes were incubated in an oven at 35 °C. Colony-forming units (CFUs) were counted after 24 h of incubation in the TSA and TCBS media and after 48 h in the MRS medium.

### 2.8. Intestinal Histomorphometry

Proximal intestine portions from the fish with an average length of 5 cm were fixed in 10% buffered formalin solution for 24 h. Subsequently, the samples were dehydrated with a progressive series of alcohol solutions, diaphanized in xylene, embedded in paraffin, and sectioned at a 5 µm thickness using a microtome, following an adaptation of the methodology in [25]. The samples were stained using the periodic acid Schiff (PAS) staining method. The slides were observed with an optical microscope (Opticam Tecnologia em Microscópios Ltd., São Paulo, Brazil) and photographed using a digital camera (Moticam 2300, 3 MP, resolution 3264 × 2448, Hong Kong, China). One histological section per sample was selected for the morphometric analysis. Villous height and width [8] were measured using the image analyzer software ToupTek ToupView—x64 version 2270/07/03. Goblet cells were quantified per villus in ten representative villi, using the same intestinal villi selected for morphometric measurements. All histomorphometric analyses were completed in a blind manner.

### 2.9. Intestinal Enzyme Activities

After euthanasia, the fish were immediately placed on ice and dissected to separate their intestines. The intestines were removed through a longitudinal incision in the belly and immediately frozen at −80 °C until analysis. For the enzymatic assays, the intestines were sectioned into small fragments and transferred to 2 mL microcentrifuge tubes where they were homogenized in ice-cold distilled water at a 1:10 ratio (*w*/*v*). The samples were then subjected to ultrasonic disruption to lyse the intestinal cells and release the digestive enzymes. Sonication was carried out in 5 cycles of 1 min, with 1 min intervals, totaling 5 min, while maintaining the samples in an ice bath. Following homogenization, the samples were centrifuged at 7000 rpm for 10 min, and the resulting supernatants were collected for enzymatic activity determination.

Amylase activity was quantified spectrophotometrically at 580 nm using 0.3% soluble starch prepared in Na_2_HPO_4_ buffer (pH 7.4) as the substrate, according to [26]. One unit of amylase activity (U) corresponded to the amount of enzyme required to hydrolyze 1 mg of starch per milliliter of enzyme extract after 30 min of incubation at 37 °C. Total alkaline protease activity was assessed following a 30 min incubation at 25 °C using 0.5% (*w*/*v*) casein dissolved in 50 mM Tris-HCl buffer (pH 8.0). The enzymatic reaction was terminated by adding 20% (*w*/*v*) trichloroacetic acid. After centrifugation at 5000 rpm for 20 min, the absorbance of the supernatant was measured at 280 nm at room temperature. Protease activity was expressed as units per milliliter, where one unit (U) represented the hydrolysis of 1 µmol of casein per minute per milliliter of enzymatic extract [27]. Lipase activity was determined by monitoring absorbance at 410 nm using p-nitrophenyl laurate (3 mM) dissolved in propanol as the substrate, following the method described in [28]. The reaction was stopped by the addition of acetone. One unit of lipase activity (U) was defined as the amount of enzyme capable of hydrolyzing 1 µmol of p-nitrophenyl laurate within 20 min at 25 °C per milliliter of enzymatic extract.

### 2.10. Statistical Analysis

Statistical analyses were performed using BioEstat software, version 1.3. Percentage values were converted (arcosine) before analysis. Normality (Shapiro–Wilk test) and homogeneity of variances (Levene test) were verified, and the data were subjected to analysis of variance (ANOVA). Data on the growth kinetics of lactic acid bacteria and pH in the fermented feeds were subjected to regression analysis. The regression model (linear or polynomial) with the best coefficient of determination was chosen to estimate the response. Productive performance data were initially analyzed using a randomized block ANOVA. As no block effect was detected (*p* > 0.05) for any parameter, the data were subsequently analyzed using a completely randomized design. When significant, the means were compared using Tukey’s test at the 5% significance level, and data were also subjected to regression analysis.

## 3. Results

### 3.1. Feed Fermentation

**Figure 1:**
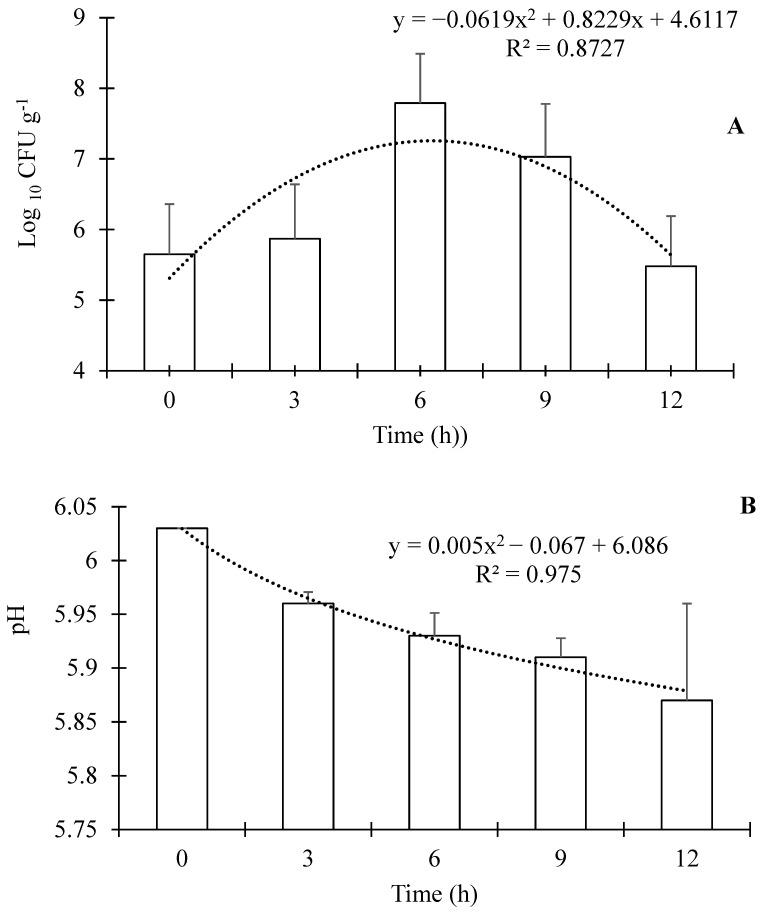
(**A**) Growth kinetics (mean ± SD *p* < 0.001) of lactic acid bacteria during the fermentation of feeds. (**B**) pH (mean ± SD *p* < 0.001) values throughout the process.

During fermentation, there was a quadratic effect (*p* < 0.0001; R^2^ = 0.8727), with the maximum lactic acid bacteria concentration in the feed occurring after 6 h of fermentation (Figure 1A). Beyond this point, the culture entered a decline phase, and the bacterial count began to decrease. The pH decreased through the fermentation time (Figure 1B), and the data also showed a better adjustment to a quadratic model (*p* < 0.0001; R^2^ = 0.9750).

### 3.2. Productive Performance

All fermented feeds resulted in lower (*p* < 0.05) feed intake compared to the control treatment, without negative effects (*p* > 0.05) on fish growth performance (Table 3). The feed intake data showed a best fit (R^2^ = 0.5438) to the quadratic model. Feed fermented for 3 and 6 h improved (*p* < 0.05) feed conversion. These data also fit (R^2^ = 0.6857) a quadratic equation, confirming improved conversion results at fermentation times of 3 and 6 h.

### 3.3. Hepatic and Muscular Ammonia

**Figure 2:**
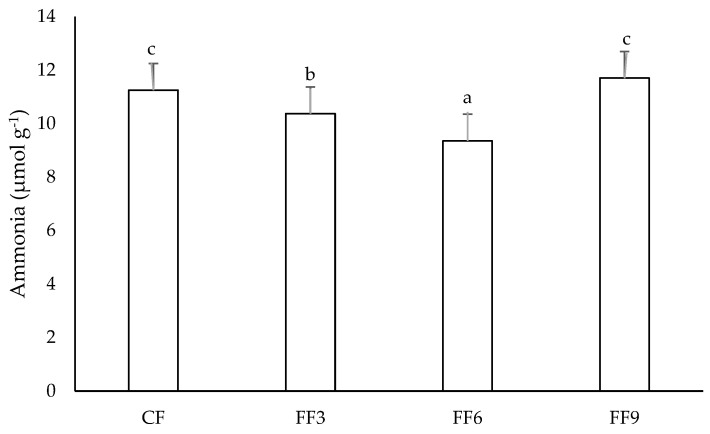
Hepatic ammonia concentrations (mean ± SD) in the juvenile rainbow trout fed fermented feeds. The means followed by different letters differ after the Tukey test (*p* < 0.0001). y = 0.0897x^2^ − 0.7959x + 11.428 R^2^ = 0.6986. CF: control feed; FF3: 3 h fermented feed; FF6: 6 h fermented feed; FF9: 9 h fermented feed.

The juvenile rainbow trout fed fermented diets showed reductions (*p* < 0.05) in hepatic ammonia concentrations (Figure 2). There was a better adjustment (R^2^ = 0.6986) to the quadratic equation, and reduction was more evident (*p* < 0.05) in the feeds fermented for 6 h. On the other hand, the ammonia concentrations in white muscle did not differ (*p* > 0.05) between the treatments (5.61 ± 1.18 µmol g^−1^).

### 3.4. Intestinal Microorganism Count

The concentration of lactic acid bacteria was higher (*p* < 0.05) in the juvenile rainbow trout fed 6 h fermented feeds compared to the other treatments (Table 4). The quadratic equation provided the best fit, but the value of R^2^ was not high (R^2^ = 0.2859).

### 3.5. Intestinal Histomorphometry

The feed fermented for 9 h increased (*p* < 0.05) the goblet cell counts in the intestines of the fish (Table 5). The results showed a better fit (R^2^ = 0.687) to the quadratic equation.

### 3.6. Intestinal Enzyme Activities

The use of fermented feeds increased (*p* < 0.05) lipase activity in the intestines of the rainbow trout (Table 6). The data had the best adjustment (R^2^ = 0.8041) to the quadratic equation, and higher activity was obtained with the feed co-fermented for 6 h.

## 4. Discussion

The successful fermentation of commercial extruded feed was confirmed by an increase in lactic acid bacteria count accompanied by a reduction in pH. The lactic acid bacteria count rapidly reached the expected value range of 7.79 CFU g^−1^ [8,29] within 6 h of fermentation. This result was consistent with a previous study using plant-based feeds for Nile tilapia (*Oreochromis niloticus*), in which peak lactic acid bacteria growth also occurred after 6 h using a similar fermentation protocol [8]. In contrast, the fermentation of feeds for largemouth bass (*Micropterus salmoides*) with *L acidophilus*, *Limosilactobacillus reuteri*, and *Lactiplantibacillus plantarum* required up to three days [7]. In the present study, the shorter fermentation time was likely due to the use of a previously activated inoculum and the physical characteristics of the feed. Extruded feed is porous and highly aerated, which may favor microbial activity and accelerate the fermentation process. The feed fermentation resulted in a slight reduction in protein, as expected. The goal of the feed fermentation was not microbial protein synthesis, but rather, partial nutrient pre-digestion to generate specific biological properties, consistent with previous reports [8,29]. Fermented feeds have also shown a reduction in free amino acids, likely because these readily available substrates are utilized by lactic acid bacteria during fermentation. After 12 h of fermentation, corresponding to the end of the exponential growth phase, a decline in lactic acid bacteria count was observed. Based on these results, fermentation times of 3, 6, and 9 h were selected for the fish trial to represent partial fermentation, peak lactic acid bacteria growth, and the onset of the decline phase, respectively.

Feeds fermented for 3 and 6 h improved the feed conversion efficiency of the juvenile rainbow trout. The positive effects on feed efficiency could be attributed to the symbiotic action of the fermented feed [7,12]. Similar results were reported for co-fermented diets used in largemouth bass [7], despite differences in fermentation methods and microbial consortia. This consistency across species and protocols strengthens the evidence that fermentation can be applied rapidly and effectively to commercial fish feeds to enhance nutrient utilization. One plausible explanation for the improved feed conversion was that fermentation may have promoted the pre-digestion of nutrients, increasing their availability and absorption [8,12]. During the fermentation, proteins are hydrolyzed into small peptides which possess specific absorption sites [30]. Accordingly, increases in soluble protein, indicative of low-molecular-weight peptides, have been reported during the fermentation of soybean meal [29] and Nile tilapia feed [8], supporting the mechanism proposed in this study.

Feeding fermented feeds reduced feed intake without impairing the growth of the rainbow trout. The effects of the fermentation on palatability and feed intake remain unclear [7,8,11], and its influence on feeding behavior still requires further investigation. In some species, such as Nile tilapia, fermented plant-based feeds with *L. acidophilus* have been shown to increase feed intake [8]. During fermentation, volatile organic compounds are produced, enhancing feed palatability and stimulating feed intake in fish [15]. Conversely, reduced feed intake associated with maintained growth has also been reported in largemouth bass, suggesting the involvement of a physiological mechanism rather than simple changes in palatability [7]. Notably, this feeding response appears to be consistent across different species and rearing conditions. Beyond direct effects on palatability, fermented feeds may influence feeding behavior through the bioactive compounds released during fermentation. Fermentation is known to generate bioactive peptides [9,10]. The potential presence of neuroactive peptides capable of modulating animal behavior cannot be excluded and warrants further investigation.

Feeding juvenile rainbow trout with the feed fermented for 6 h reduced hepatic ammonia concentrations. This result suggests more efficient protein utilization and supports the observed improvements in feed conversion. Hepatic ammonia is generated primarily through amino acid deamination, which occurs when dietary protein is catabolized rather than directed toward growth or other anabolic functions. Therefore, lower hepatic ammonia levels indicate a metabolic shift toward protein retention and tissue synthesis. Fermentation promotes partial protein hydrolysis mediated by microbial and endogenous enzymes, generating low–molecular-weight peptides [8,29] that are absorbed more efficiently than intact proteins due to specialized uptake mechanisms in the intestinal epithelium. Consistent with this mechanism, diets containing hydrolyzed proteins have also been shown to reduce hepatic ammonia levels in South American catfish (*Rhamdia quelen*) [30]. Further assessment of protein digestibility and nitrogen retention in fermented diets is needed to better understand the effects of fermentation on protein absorption.

Feed fermented for 6 h increased the concentration of acid lactic bacteria in the intestines of the juvenile rainbow trout. Fermented feeds are recognized as natural sources of microorganisms with probiotic potential [8,11,12]. During the growth kinetics of lactic acid bacteria, the highest bacterial concentration was observed after six hours of fermentation. This increased microbial load in the feed likely contributed to enhanced gut colonization. Similarly, the ingestion of plant-based fermented feed also increased the count of lactic acid bacteria in Nile tilapia [8]. The increased intestinal concentration in lactic acid bacteria may also have been associated with improvements in feed conversion in the juvenile rainbow trout. The positive effects of probiotic microorganisms on feed efficiency in fish are well established [31,32,33]. Although fermented feed and ingredients can reduce *Vibrio* counts in fish intestines [8,29], no *Vibrio* growth was detected in any of the treatments. This result was possibly due to the cold-water rearing conditions [34,35].

The juvenile rainbow trout fed feed fermented for 9 h exhibited an increased number of intestinal goblet cells, indicating improved intestinal condition. Goblet cells secrete mucins that form the intestinal mucus layer, which lubricates the lumen and protects the epithelium from mechanical and chemical damage [36]. This mucus layer also plays a key role in maintaining gut barrier function, as it regulates mucus composition and contributes to host defense against pathogens. Similar increases in goblet cell numbers have been observed in juvenile Nile tilapia fed fermented plant-based feeds [8]. The symbiotic effect of fermented feed can explain the positive impacts on intestinal histomorphometry [11,12]. Notably, no increases in goblet cell counts were observed in the fish fed feed fermented for 6 h, despite this treatment having the highest concentration of lactic acid bacteria. Fermentation time modulates the microbial and biochemical profiles of fermented feeds [8], which, in turn, can differently influence physiological responses in fish.

The ingestion of feed fermented for 6 h increased intestinal lipase activity. This finding aligned with the improvements observed in feed conversion and the increased concentrations of intestinal lactic acid bacteria, and it may be associated with enhanced feed efficiency and intestinal condition. During feed fermentation, lactic acid bacteria produce enzymes that are released into the medium and contribute to nutrient breakdown [37,38]. In addition, the higher intestinal colonization by lactic acid bacteria in the fish fed this feed may also have contributed to the increased lipase activity observed. Previous studies have shown that the ingestion of fermented ingredients such as soybean meal can improve intestinal enzyme activity [39]. In contrast, no changes in intestinal enzyme activity were reported in juvenile Nile tilapia fed fermented plant-based feed [8].

## 5. Conclusions

The fermentation of extruded commercial feed with *L. acidophilus* and *L. rhamnosus* was fast and efficient, as demonstrated by the increase in lactic acid bacteria and the reduction in pH after 6 h of fermentation.

Feeding rainbow trout juveniles with fermented feeds reduced feed intake without affecting fish growth performance. Feed fermented for 3 and 6 h improved feed efficiency, and evidence of enhanced nitrogen utilization was observed. In addition, feed fermented for 6 h increased intestinal colonization by acid lactic bacteria and enhanced intestinal lipase activity. Feed fermented for 9 h showed evidence of improved intestinal condition. Overall, this study indicates that feed fermentation improves feed efficiency and intestinal condition in rainbow trout.

## Figures and Tables

**Table 1:** Centesimal composition of the fermented feeds.

Constituent	Treatments			
	CF	FF3	FF6	FF9
Dry matter	94.58 ± 0.09	95.10 ± 0.05	94.77 ± 0.10	93.61 ± 0.07
Ether extract	9.96 ± 0.40	10.13 ± 0.36	10.34 ± 0.28	10.74 ± 0.09
Crude protein	49.84 ± 0.11	48.42 ± 0.17	48.81 ± 0.19	48.56 ± 0.34
Ash	10.24 ± 0.01	10.03 ± 0.01	10.02 ± 0.04	10.02 ± 0.03
Insoluble fiber	2.27 ± 0.08	2.06 ± 0.16	1.88 ± 0.06	1.51 ± 0.04
Soluble fiber	27.70 ± 1.51	32.91 ± 0.52	32.90 ± 0.89	26.89 ± 2.16
GE/CP	9.87	9.59	9.67	10.18
GE (kcal g^−1^)	4.92	4.65	4.72	4.94

GE/CP: gross energy and crude protein ratio; GE: gross energy; CF: control feed; FF3: 3 h fermented feed; FF6: 6 h fermented feed; FF9: 9 h fermented feed.

**Table 2:** Free amino acid composition of the fermented feeds (g kg^−1^ dry matter).

Amino Acids	Treatments			
	CF	FF3	FF6	FF9
Aspartate	0.010 ± 0.00	0.002 ± 0.00	0.010 ± 0.00	0.008 ± 0.0012
Glutamate	Ud	0.037 ± 0.001	Ud	0.046 ± 0.0014
Asparagine	0.024 ± 0.001	0.020 ± 0.001	0.021 ± 0.001	0.023 ± 0.0011
Serine	0.012 ± 0.00	0.011 ± 0.00	0.005 ± 0.00	0.008 ± 0.006
Glutamine	0.003 ± 0.00	0.003 ± 0.00	Ud	0.003 ± 0.00027
Histidine	Ud	Ud	Ud	Ud
Glycine	Ud	Ud	Ud	Ud
Threonine	0.043 ± 0.004	0.041 ± 0.003	0.043 ± 0.00	0.056 ± 0.002
Arginine	0.043 ± 0.003	0.042 ± 0.003	0.047 ± 0.008	0.077 ± 0.001
Alanine	0.204 ± 0.004	0.202 ± 0.002	0.18 ± 0.034	0.160 ± 0.001
Tyrosine	0.043 ± 0.001	0.044 ± 0.005	0.044 ± 0.008	0.041 ± 0.003
Cysteine	0.005 ± 0.00	0.005 ± 0.00	0.007 ± 0.002	Ud
Valine	Ud	0.011 ± 0.001	0.016 ± 0.001	0.053 ± 0.001
Methionine	0.856 ± 0.002	0.733 ± 0.010	0.775 ± 0.041	0.768 ± 0.009
Tryptophan	Ud	Ud	Ud	Ud
Phenylalanine	0.45 ± 0.01	0.044 ± 0.001	0.027 ± 0.006	0.009 ± 0.00
Isoleucine	0.115 ± 0.003	Ud	0.126 ± 0.017	0.147 ± 0.001
Leucine	0.251 ± 0.003	0.223 ± 0.003	0.218 ± 0.001	0.245 ± 0.004
Lysine	0.611 ± 0.001	0.495 ± 0.003	0.557 ± 0.007	0.533 ± 0.0021

Limit of quantification (LQ): 0.002 ± 0.1 mg amino acid g^−1^ of dry matter; Ud: undetected/under the quantification limit; CF: control feed; FF3: 3 h fermented feed; FF6: 6 h fermented feed; FF9: 9 h fermented feed.

**Table 3:** Productive performance (mean ± SD) of the rainbow trout fermented feeds.

Parameters	Treatments				
	CF	FF3	FF6	FF9	*p*-Value
Initial weight (g)	21.88 ± 1.03	22.08 ± 0.33	22.12 ± 1.28	22.79 ± 0.76	0.6215
Final weight (g)	60.52 ± 6.07	65.07 ± 3.85	62.16 ± 3.42	58.54 ± 3.92	0.2529
Daily weight gain (g)	0.64 ± 0.0	0.72 ± 0.07	0.67 ± 0.05	0.60 ± 0.07	0.1582
Daily feed intake (g) *	1.02 ± 0.08 a	0.91 ± 0.05 b	0.89 ± 0.03 b	0.87 ± 0.06 b	0.0176
Feed conversion rate **	1.56 ± 0.06 b	1.28 ± 0.09 a	1.33 ± 0.10 a	1.49 ± 0.07 ab	0.0014
Specific growth rate (%.day^−1^)	1.44 ± 0.58	1.80 ± 0.12	1.72 ± 0.11	1.57 ± 0.15	0.4017
Hepatosomatic index (%)	2.07 ± 0.28	2.10 ± 0.26	2.14 ± 0.61	2.19 ± 0.23	0.9512
Survival (%)	98.3 ± 3.35	98.3 ± 3.35	98.3 ± 3.35	96.6 ± 3.87	0.8804

The means followed by different letters differed after the Tukey test (*p* < 0.05). CF: control feed; FF3: 3 h fermented feed; FF6: 6 h fermented feed; FF9: 9 h fermented feed. * y = 0.0025x^2^ − 0.038x + 1.0145 R^2^ = 0.5438; ** y = 0.0124x^2^ − 0.1175x + 1.5593 R^2^ = 0.6857.

**Table 4:** Intestinal microorganism counts (mean ± SD CFU Log 10 g^−1^) of the rainbow trout fed fermented feed.

Parameters	Treatments				
	CF	FF3	FF6	FF9	*p*-Value
Heterotrophic bacteria	7.17 ± 0.68	7.19 ± 0.69	7.42 ± 0.32	7.44 ± 0.33	0.5336
Lactic acid bacteria *	5.03 ± 0.13 b	5.08 ± 0.28 b	5.50 ± 0.27 a	5.17 ± 0.10 b	0.0311
*Vibrio* sp.	ND	ND	ND	ND	-

The means followed by different letters differed after the Tukey test (*p* < 0.05). CF: control feed; FF3: 3 h fermented feed; FF6: 6 h fermented feed; FF9: 9 h fermented feed; ND: not detected. * y = 0.0105x^2^ − 0.1204x + 4.9897 R^2^ = 0.2859.

**Table 5:** Intestinal histomorphometry (mean ± SD) of the rainbow trout fed fermented feed.

Parameters	Treatments				
	CF	FF3	FF6	FF9	*p*-Value
Villus height (µm)	429.51 ± 100.19	459.14 ± 84.49	399.25 ± 68.96	439.75 ± 44.80	0.7433
Villus width (µm)	135.79 ± 13.31	113.42 ± 10.66	111.79 ± 9.89	99.37 ± 14.02	0.1518
Goblets cells *	7.56 ± 2.20 b	7.95 ± 1.16 b	7.77 ± 1.47 b	11.02 ± 2.05 a	0.0068

The means followed by different letters differed after the Tukey test (*p* < 0.05). CF: control feed; FF3: 3 h fermented feed; FF6: 6 h fermented feed; FF9: 9 h fermented feed. * y = 0.1751x^2^ − 1.0304x + 7.517 R^2^ = 0.687. Goblet cell samples were counted individually, and the results were expressed as number of cells per villus.

**Table 6:** Specific activity of the digestive enzymes (means ± SD Ug tissue^−1^) of the juvenile rainbow trout (*Oncorhynchus mykiss*) fed fermented diets.

Parameters	Treatments				
	CF	FF3	FF6	FF9	*p*-Value
Amylase	1.01 ± 0.41	1.38 ± 1.15	1.33 ± 0.86	1.02 ± 0.33	0.0814
Lipase *	2.53 ± 0.29 c	3.40 ± 0.17 b	4.06 ± 0.38 a	3.55 ± 0.23 ab	0.0010
Total alkaline protease	0.14 ± 0.02	0.16 ± 0.04	0.19 ± 0.05	0.17 ± 0.10	0.0512

The means followed by different letters differed after the Tukey test (*p* < 0.05). CF: control feed; FF3: 3 h fermented feed; FF6: 6 h fermented feed; FF9: 9 h fermented feed. * y = −0.0386x^2^ + 0.4717x + 2.4777 R^2^ = 0.8041.

## Data Availability

The data that support this study’s findings can be obtained from the corresponding author upon reasonable request.
